# Finding potential cis-regulatory loci using allele-specific chromatin accessibility as weights in a kernel-based variance component test

**DOI:** 10.1186/s12919-016-0013-1

**Published:** 2016-10-18

**Authors:** Juan Manuel Peralta, Marcio Almeida, Lawrence J. Abraham, Eric Moses, John Blangero

**Affiliations:** 1 South Texas Diabetes and Obesity Institute, University of Texas at the Rio Grande Valley, One West University Blvd., Brownsville, TX 78520 USA; 2 South Texas Diabetes and Obesity Institute, University of Texas Health Science Center, 7703 Floyd Curl Drive, San Antonio, TX 78229 USA; 3 Centre for Genetic Origins of Health and Disease, University of Western Australia, 35 Stirling Hwy, Crawley, WA 6009 Australia

## Abstract

We present a novel approach to detect potential *cis*-acting regulatory loci that combines the functional potential, an empirical DNase-seq based estimate of the allele-specificity of DNase-I hypersensitivity sites, with kernel-based variance component association analyses against expression phenotypes. To test our method we used public ENCODE whole genome DNase-I sequencing data, from a single sample, to estimate the functional potentials of the subset of 10,552 noncoding heterozygous single-nucleotide polymorphisms (SNPs) that were also present in the Genetic Analysis Workshop 19 (GAW19) family-based data set. We then built two covariance kernels, one nonweighted and one weighted by the functional potentials, and conducted kernel-based variance component association analyses against the 20,527 transcript expression phenotypes in the GAW19 family-based data set. We found signals of potential *cis*-regulatory effects, that surpassed the Bonferroni significance threshold, for ten transcripts. Stepwise removal of the *cis*-located SNPs from the weighted kernel lead to the disappearance of the association signal from our top transcript hit. We found compelling evidence of allele-specific *cis*-regulation for four transcripts using both kernels, and our results agree with previous research that suggests the involvement of specific *cis*-located variants in the regulation of their neighboring gene.

## Background

Variation found in noncoding regions of the genome is much more abundant and, perhaps, even more relevant than coding variation for certain human traits, but its biological meaning is hard to assess [1]. It has been noticed that between 34 and 88 % of the disease-associated variants detected by genome-wide association studies (GWAS) appear to cluster in noncoding regions of the genome, specifically in DNase-I hypersensitivity sites (DHSs) [2], and that some of the DHSs exhibit allele-specificity [2–4]. Chromatin remodeling processes, for example those associated with the transcription machinery, create openings in the chromatin, which can be detected as DHSs, that allow transcription factors to interact with the underlying DNA. Hence DHSs tend to correlate with known *cis*-acting regulatory elements, such as promoters and transcription factor binding sites [5].

We have been investigating a systematic approach that uses DHSs to determine if noncoding single-nucleotide variation changes the local allele-specific chromatin accessibility, something that would indicate a potential regulatory role for a variant [6]. We have also developed a variance component based burden test to determine the contribution of localized relationship kernels to the trait variance [7, 8]. Here, we test if by combining both lines of research we could detect potential *cis*-acting regulatory loci. Our approach differs from previous works [4, 9] in that (a) we evaluate the association of each expression phenotype against a single covariance kernel, in a 1 degree of freedom test, and (b) we use an allele-specific chromatin accessibility measure to filter and weight the variants.

## Methods

### Data set

We used single-nucleotide polymorphism (SNP) dosages from 959 genotyped individuals, transcript expression levels from 647 of those individuals, and the genealogies (1389 individuals in 20 families) that were provided as part of the Genetic Analysis Workshop 19 (GAW19) family-based data set [10]. In addition, we used publicly available data from a CEU-CEPH (Northern Europeans from Utah–Centre d’Etude du Polymorphisme Humain) female’s peripheral blood mononucleated cells, NA12878, and its derived lymphoblastoid cell line, GM12878. The specific data used were: whole genome sequencing (WGS) genotypes for NA12878, from Illumina’s Platinum Genomes [11], and mapped short-sequencing reads (reads) from all five replicates of the DHSs sequencing (DNase-seq) of GM12878, from ENCODE [12], were used in this study. Physical coordinates and annotations for genes, transcripts, and marker loci refer to release 19 of the human genome (hg19) from the University of California, Santa Cruz (UCSC).

### Reference panel of heterozygous single-nucleotide polymorphism loci

We compiled a reference panel of heterozygous SNP sites from the genotype calls from the high-coverage/high-quality WGS of NA12878. This independent genotypes source allowed us to analyze heterozygous loci where, because of either low coverage or complete allele-specific accessibility, only 1 allele is represented in the DNase-seq reads.

### Chromatin accessibility measurement

We defined our chromatin accessibility measure to be equal to the DNase-seq read depth of each allele at a heterozygous locus. Based on our previous experience [6] the DNase-seq reads from all five GM12878 replicates were pooled to increase the total sequencing coverage at the DHSs. Samtools [13] mpileup was then used to obtain genotype calls only for loci in the known NA12878 heterozygous reference panel, and allele-specific read depths were obtained from the count of forward and reverse mapped reference and alternative allele annotations stored in the DP4 tag of the generated variant call format (VCF) file.

### Functional potential

A departure from the expectation of an equal chromatin accessibility measurement of the two alleles at a locus within a DHS is what we refer to as the locus functional potential (FP). We implemented the FP statistic as a likelihood ratio–based test that contrasts the observed allele read depths with their expected depth at known heterozygous loci within DHSs [6]. A significant bias toward 1 allele in the chromatin accessibility measure of a locus can indicate a putative allele-specific chromatin remodeling event that compromised the footprint left by a DHS. We estimated the FP for all known NA12878 heterozygous loci that were present in the DNase-seq of GM12878.

### Trait and covariates

To test our approach we used the real expression phenotypes from approximately 20,000 transcripts provided in the GAW19 family data set [10]. In addition, we simulated 10,000 heritable quantitative phenotypes not associated with any of the SNP loci in the data set, using Sequential Oligogenic Linkage Analysis Routines (SOLAR) [14], to evaluate the performance of our test under a null hypothesis.

We also used the sex, age, their interactions, and the smoking status at the first visit as covariates in all models. The first two principal components (PC1, PC2) (estimated as described in Peralta et al. [7] and Almeida et al. [8]), were added to account for any unknown population substructure that might be present.

### Covariance kernels

GAW19 SNP dosages were collected for all heterozygous loci from NA12878 with a FP estimate. Non informative loci were removed. A standardized dosages matrix, **Z**, was built from them, and the covariance matrix of the dosages, **R**, was obtained from

$$ \boldsymbol{R}=\boldsymbol{Z}\cdot {\boldsymbol{Z}}^T $$

The covariance matrix was then scaled so that all diagonal elements were equal to 1, and the resulting matrix, **K**, was our nonweighted covariance kernel.

We also built a covariance kernel in which each locus contribution was weighted by its FP estimate. Because our FP statistic is a likelihood ratio test, we used the relative − loglikelihood from a locus against the sum of all loci − loglikelihoods as the locus weight, and thus all weights add up to 1. The covariance kernel, **K,** was constructed as before, with 1 exception. The covariance matrix of the dosages was obtained from

$$ \boldsymbol{R}=\boldsymbol{Z}\cdot {\boldsymbol{D}}_w\cdot {\boldsymbol{Z}}^T $$

where ***D***_**w**_ is a diagonal matrix of weights.

### Variance component model

We used the variance component model previously described in Peralta et al. [7] and Almeida et al. [8], in conjunction with the nonweighted and FP-weighted covariance kernels derived from the SNP dosages described above, to estimate the proportion of the phenotypic variance, *h*_*geff*_^2^, explained by allele-specific genetic variants found within DHSs in an unrelated CEU-CEPH individual. The *h*_*geff*_^2^ variance component, and its significance, was estimated for each real and simulated expression phenotype using SOLAR, a flexible genetic variance component analysis program with a focus on general pedigrees [14].

## Results

Our reference panel of heterozygous loci contained the 2,423,308 heterozygous SNPs that had been found in the WGS of NA12878. Only heterozygous loci are informative for allele-specific chromatin accessibility in a genome. Although heterozygous SNP sites can be directly inferred from DNase-seq data, it is not ideal, in part because of its very low coverage.

We were able to measure the allele-specific chromatin accessibility and estimate the FP for 48,236 (1.99 %) of those heterozygous SNPs but only 10,618 (22 %) of them were present in the GAW19 dosages. Of the 10,618 heterozygous-in-NA12878 SNPs with a FP estimation that were present in GAW19, 66 (0.62 %) were monomorphic in the GAW19 dosages and were therefore discarded from further analysis. The remaining 10552 SNPs with FP estimates were used for the construction of our weighted and nonweighted covariance kernels.

**Fig. 1:**
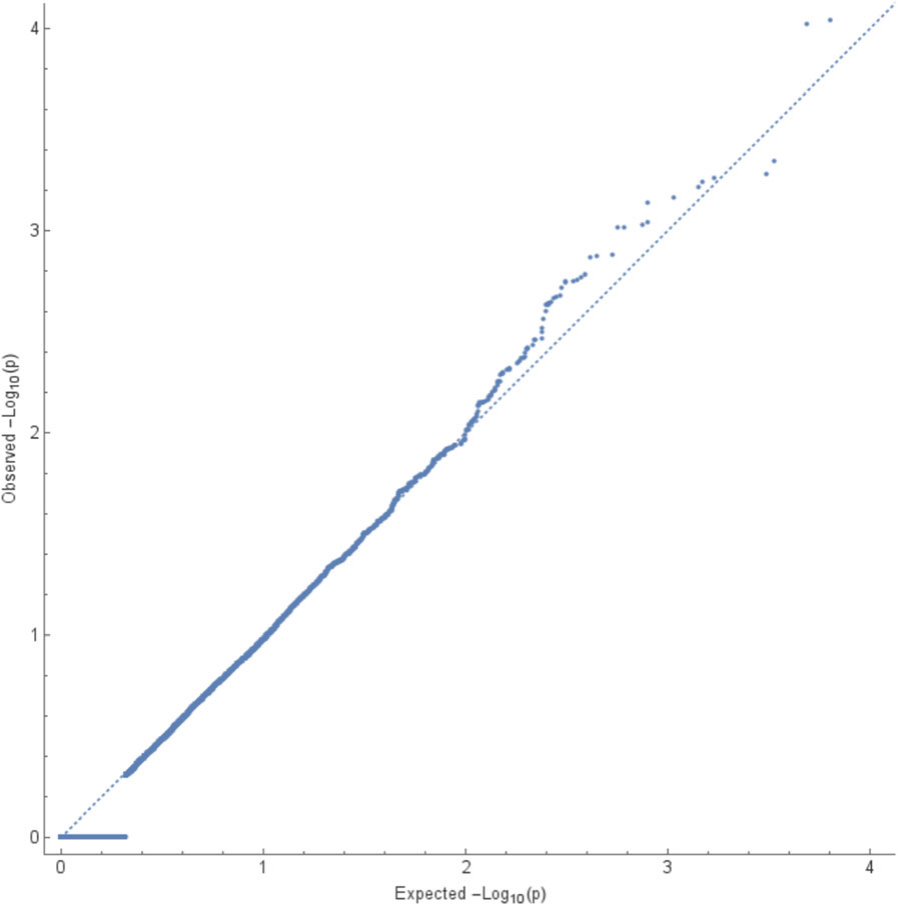
Quantile–quantile (Q-Q) plot of the *p* values obtained under a null hypothesis test. Analysis of 10,000 simulated phenotypes not associated with any of the GAW19 SNP loci. The obtained the *p* values follow the expected uniform distribution

**Fig. 2:**
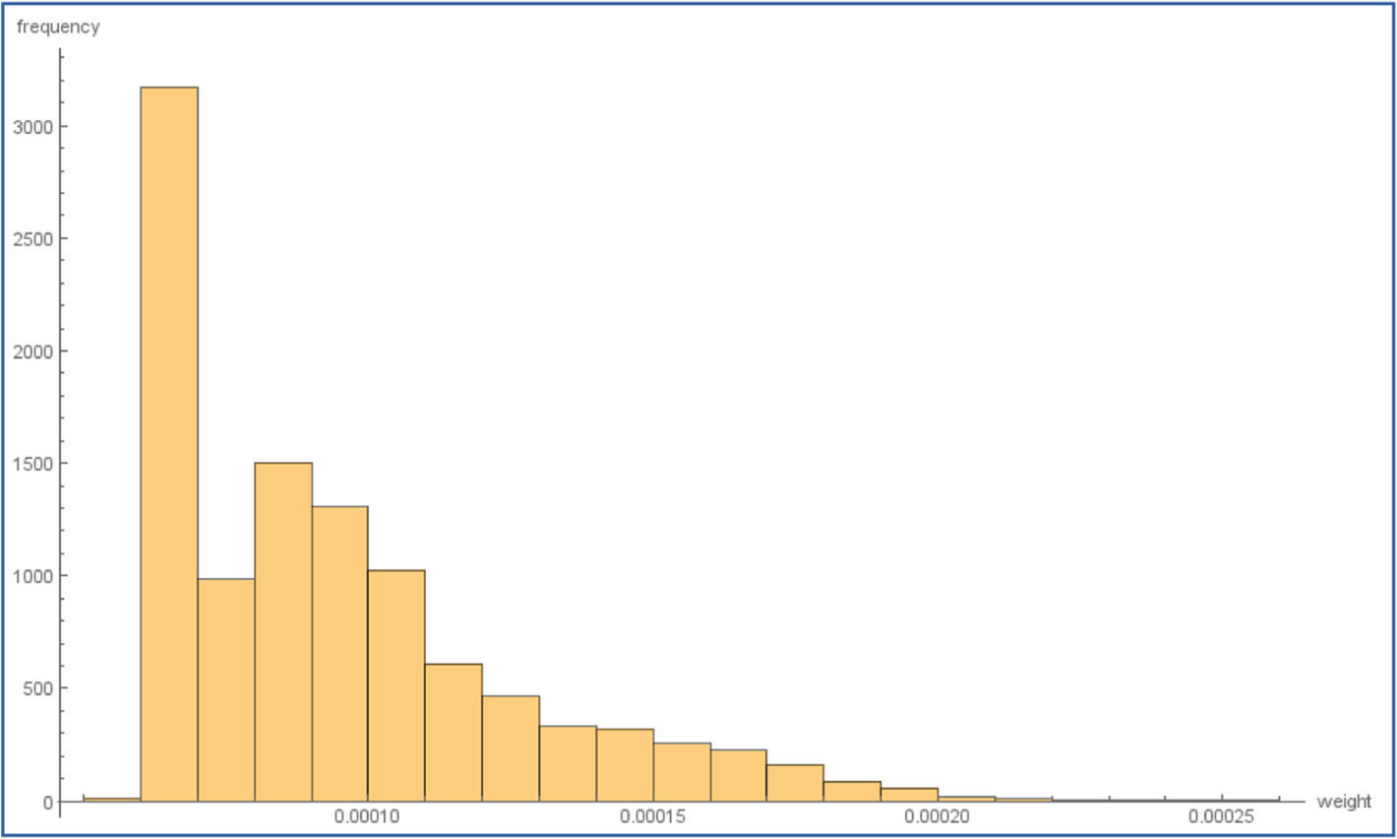
Frequency distribution of the weights used for variants in the weighted covariance kernel. Each weight represents the relative proportion of the functional potential − loglikelihood estimation of each variant in the kernel. The large proportion of variants in the first bin have a very small weight, and correspond to variants with a low confidence of having an allele-specific chromatin accessibility effect

We conducted our variance component analysis of 10,000 simulated phenotypes using the weighted covariance kernel only and found no inflation or deflation of the *p* values of the estimated effects (Fig. 1), indicating that our test performed as expected when evaluated under the null hypothesis. Figure 2 shows the frequency distribution of the weights.

We then analyzed the 20,527 transcript expression phenotypes in the GAW19 family data set using both the weighted and the nonweighted covariance kernels. After a genome-wide Bonferroni correction (−log_10_[α] = 5.6) we found significant evidence of potential *cis*-regulatory effects for ten transcripts (Table 1). Eight of the transcripts were detected by both covariance kernels but two of them, GI_4506738-S and GI_15451941-S, were only found to be significant when the weighted covariance kernel was used. In most of the cases, the use of the nonweighted covariance kernel tended to slightly decrease the proportion of the transcript expression variance explained by the kernel, which was on average very high in both cases (*h*_*geff*,*non* − *weighted*_^2^ = 0.6540, *h*_*geff*, *weighted*_^2^ = 0.7046). While most of the trait heritability was explained by the covariance kernel, a substantial amount (between 14 and 28 %) still remained. Table 2 lists these ten transcripts along with their annotations and closest SNPs in the covariance kernels. Table 3 shows how the signal from our top result, GI_42544126-I, decreases when SNPs within the transcript region are progressively removed from the kernel.

**Table 1:** Transcripts for whom their variation in expression levels can be explained by a covariance kernel composed by SNP with FP estimates, at genome-wide significance

Transcript	Covariance kernel							
	Non-weighted				Weighted			
	h2r	h2r_p	geff	geff_p	h2r	h2r_p	Geff	geff_p
GI_42544126-I	0.0000	0.5000	0.7074	4.03E-15	0.0000	0.5000	0.7145	4.55E-18
GI_23097237-S	0.0000	0.5000	0.7848	1.15E-14	0.0000	0.5000	0.7493	7.46E-12
GI_10863968-S	0.0000	0.5000	0.6109	5.68E-10	0.0000	0.5000	0.6122	9.05E-11
Hs.283934-S	0.0746	0.3138	0.8382	9.77E-10	0.1497	0.1443	0.7657	4.87E-09
GI_12056480-A	0.2357	0.0457	0.7069	1.69E-08	0.2792	0.0194	0.6628	5.43E-08
GI_20986517-S	0.0000	0.5000	0.7671	5.58E-08	0.0000	0.5000	0.7726	3.08E-08
Hs.58104-S	0.2230	0.0753	0.6886	6.89E-07	0.2705	0.0333	0.6415	8.47E-07
GI_41393558-I	0.0000	0.5000	0.5331	1.92E-06	0.0000	0.5000	0.5371	1.73E-06
GI_4506738-S	NA				0.0000	0.5000	0.4758	6.66E-07
GI_15451941-S	NA				0.2611	0.0441	0.6090	1.33E-06

geff, Gene-specific effect estimate (*h* ^2^_geff_)

geff_p, significance of the gene-specific effect estimate

h2r, trait heritability estimate (*h* ^2^)

h2r_p, significance of the trait heritability estimate

**Table 2:** Annotated transcript and SNP table

Transcript	Gene	Chromosome	Start	Length	SNP	DBSnp rs	SNP annotation
GI_42544126-I	SF1	chr11	64532075	14241	11_64511322	rs2073798	RASGRP2 intron
					11_64519345	rs686171	PYGM intron
					11_64546106	rs3741398	SF1 2 kb upstream, nc transcript variant, 5’ UTR
					11_64546257	rs1633462	SF1 2 kb upstream, nc transcript variant, 5’ UTR
					11_64573589	rs669976	MEN1 intron
					11_64576598	rs67808744	MEN1 intron
					11_64577620	rs7949944	MEN1 5’ UTR, 2 kb upstream
GI_23097237-S	CHST13	chr3	126243130	19004	3_126218788	rs6774768	UROC1 intron
					3_126228953	rs1873388	UROC1 intron
					3_126242964	rs1388096	CHST13 2 kb upstream
					3_126245956	rs4592980	CHST13 intron/3’UTR
					3_126246370	rs1994642	CHST13 intron/3’UTR
					3_126247795	rs11717719	CHST13 intron
					3_126247848	rs11718493	CHST13 intron
GI_10863968-S	POLD4	chr11	67119018	2034	11_67196237	rs1476792	
Hs.283934-S	TSPAN16	chr19	11406815	30857	19_11340057	rs17001244	
					19_11358700	rs4804579	
					19_11374675	rs416231	
					19_11380295	rs4804159	
					19_11406952	rs374409	
GI_12056480-A	UTS2	chr1	7907271	6280	1_7710810	rs58905635	CAMTA1 intron
					1_7725855	rs4908471	CAMTA1 intron
					1_7749807	rs3124797	CAMTA1 intron
GI_20986517-S	MAPK8IP1	chr11	45907046	20970	11_45838926	rs11038668	
					11_45840939	rs7112505	
					11_45891418	rs7123390	CRY2 intron
Hs.58104-S	FAM101B	chr17	289771	8960	17_185027	rs12951437	
					17_198698	rs11869174	
					17_206962	rs11657163	
GI_41393558-I	KIF1B	chr1	10270763	97892	1_10270386	rs3828081	KIF1B 2 kb upstream
					1_10307453	rs4240911	KIF1B intron
					1_10438687	rs1536262	KIF1B 3’UTR
GI_4506738-S	RPS6KB2	chr11	67195934	6945	11_67196237	rs1476792	RPS6KB2 intron
					11_67204342	rs12787021	PTPRCAP intron
					11_67213956	rs2109123	
					11_67253564	rs7110021	
					11_67258805	rs751567	
					11_67264679	rs2276120	
GI_15451941-S	UBA52	chr19	18682613	5657	19_18499151	rs1059022	
					19_18499238	rs1804826	
					19_18715154	rs72995445	CRLF1 intron
					19_18859680	rs11085244	

Gene symbols and coordinates for the ten transcripts that were detected as being potentially *cis*-regulated by SNPs in our covariance kernel. The closest SNPs to each gene are listed

**Table 3:** Decrease in the association signal when *cis*-located SNPs are removed from the kernel

Transcript	Gene	SNPs removed from the kernel	Covariance kernel		
			Weighted		
			h2r	geff	geff_p
		none	0.0000	0.7145	4.55E-18
GI_42544126-I	SF1	2 in SF1	0.0000	0.6809	1.32E-12
		all in transcript region	0.1349	0.1349	2.00E-05

## Discussion

The objective of this study was to investigate the prioritization of SNPs based on their potential as functional, *cis*-acting, regulatory elements. To that end we used a combined approach that integrates functional information, in the form of allele-specific chromatin accessibility measurements at DHSs, gene expression phenotypes, and a variance component model that estimates the proportion of a trait’s variance as a result of a localized relationship kernel.

We constructed nonweighted and weighted covariance kernels, using the 10,552 SNPs with an available FP estimate, and obtained the proportion of variance in the levels of transcript expression that could be explained by them in the family data set. We identified a clear signal for eight transcripts when using the nonweighted kernel, and for two additional transcripts when using the weighted kernel (see Table 1). In contrast, we found no signals when we performed our analysis using the set of 10,000 simulated phenotypes; an indication that our test statistic was not artificially inflated when evaluated under the null hypothesis (see Fig. 1).

Some of our results are difficult to interpret because of the distance between the transcript location and the closest SNPs to it in our kernels. For transcripts GI_12056480-A and GI_15451941-S our results might indicate the presence of long-acting *cis*-elements, but could also be the result of, for example, linkage disequilibrium with SNPs in closer proximity to the transcript.

However, close examination of the annotations of the significant transcripts in our results shows suggestive evidence of potential *cis*-acting variants. Particularly for the GI_23097237-S, GI_42544126-I, GI_4506738-S, and GI_41393558-I transcripts, corresponding to the *CHST13*, *SF1*, *RP56KB2*, and *KIF1B* genes, respectively. The SNPs with FP estimates that we incorporated in our covariance kernel near these genes are all located either within the gene or within the promoter region of the gene (see Table 2). The progressive removal of SNPs within and near the *SF1* gene led to the degradation of the signal from the GI_42544126-I transcript (see Table 3), clearly suggesting a *cis*-acting effect of the variants in the transcript expression. Furthermore, previous research provides additional compelling evidence for the implication of rs11718493 in the allele-specific methylation of CpGs and the regulation of *CHST13* [15, 16], a carbohydrate sulfotransferase that is present in the Golgi membrane [17], and rs1536262 has been reported to be a likely candidate for the regulation of *KIF1B* expression [18].

## Conclusions

Our kernel-based variance component test was able to prioritize noncoding variation from whole-genome sequencing data based on their potential to regulate gene expression. An allele-specific chromatin accessibility measure was used as both a biologically meaningful filter for the selection of the variants and the weight of each variant in the covariance kernel. We observed compelling evidence to support the idea that four genes might be *cis*-regulated by the SNPs we identified in them.
